# Bioinspired flexible electronics for seamless neural interfacing and chronic recording

**DOI:** 10.1039/d0na00323a

**Published:** 2020-06-16

**Authors:** Hongbian Li, Jinfen Wang, Ying Fang

**Affiliations:** CAS Key Laboratory for Biomedical Effects of Nanomaterials and Nanosafety, CAS Center for Excellence in Nanoscience, National Center for Nanoscience and Technology Beijing 100190 China fangy@nanoctr.cn; CAS Center for Excellence in Brain Science and Intelligence Technology, Institute of Neuroscience, Chinese Academy of Sciences Shanghai 200031 China

## Abstract

Implantable neural probes are among the most widely applied tools for the understanding of neural circuit functions and the treatment of neurological disorders. Despite remarkable progress in recent years, it is still challenging for conventional rigid probes to achieve stable neural recording over long periods of time. Recently, flexible electronics with biomimetic structures and mechanical properties have been demonstrated for the formation of seamless probe–neural interfaces, enabling long-term recording stability. In this review, we provide an overview of bioinspired flexible electronics, from their structural design to probe–brain interfaces and chronic neural recording applications. Opportunities of bioinspired flexible electronics in fundamental neuroscience and clinical studies are also discussed.

## Abstract 2

This review summarizes recent developments in bioinspired flexible electronics for seamless neural interfacing and chronic recording.

## Graphical abstract

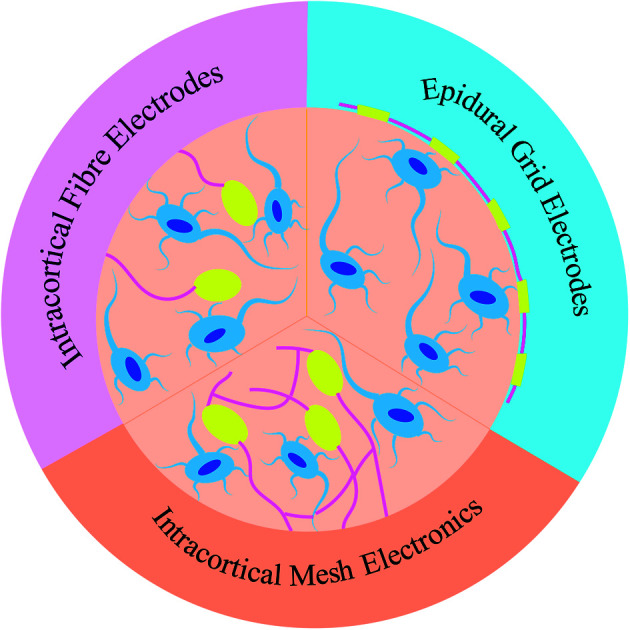

## 1. Introduction

Brain functions arise from concerted activity of large populations of neurons.^1^ During neuronal activity, the inward and outward flows of ions (Na^+^, K^+^, Ca^2+^*etc.*) through transmembrane channels give rise to ionic currents.^2^ Implantable neural probes that transduce these ion currents into extracellular potential signals are among one of the most widely applied tools to record neural activity.^3^ In particular, Utah array,^4^ Michigan array,^5^ and microwire electrodes^6^ can record neural activity at single-cell and single-spike resolution, and have greatly advanced our understanding of the underlying mechanisms of neural computation. In addition, implantable neural probes have also been applied for the treatment of neurological diseases such as Parkinson's diseases, Alzheimer's diseases, and epilepsy,^7,8^ as well as the control of neural prosthetics.^9^ However, conventional implantable probes are constructed with rigid materials, such as silicon and metals,^10^ whose Young's moduli are several orders of magnitude higher than that of soft brain tissues. This large mechanical mismatch causes the micromotion of implanted rigid probes relative to neurons of interest,^11^ resulting in signal instability with time. Moreover, the shear stress generated by the micromotion induces chronic tissue inflammatory responses and glial scar formation. Glial scars eventually encapsulate the microelectrodes as an insulation layer and lead to device performance degradation or loss.^12,13^

Over the past decades, many research efforts have been made in developing tissue-compliant flexible electronics for stable neural interfaces. For example, neural electrodes integrated on soft polymer films have been shown to elicit reduced inflammatory responses compared to their rigid counterparts.^14,15^ However, the stiffness of these planar probes is still orders of magnitude higher than that of brain tissues.^10,16^ Moreover, implanted polymer film-based planar probes can disrupt endogenous connections in neural networks,^13,17^ which precludes their seamless integration with brain tissues. In order to achieve a stable neural interface, electrode materials should have the following structural and functional properties: (i) stable electrical properties, including low impedance for high signal-to-noise ratio recording; (ii) tissue-like softness and flexibility; and (iii) high biocompatibility.^18^ Recently, bioinspired flexible electronics have been attracting increasing interests for neural interfacing because of their structural and mechanical similarity with the brain tissue.^13,19,20^ Distinct from conventional planar probes, these bioinspired flexible electronics can form three-dimensional (3D) and seamless interfaces with brain tissues and thus allow for stable neural recording over extended periods of time. In this review, we summarize recent developments of bioinspired flexible electronics that enable long-term stable chronic recording, with an emphasis on their biomimetic design and seamless bio-integration. Bioinspired flexible electronics with various structural designs and their interfaces to a number of biological systems, including synthetic tissues, spheroids/organoids, and *in vivo* brain tissues, are discussed. At last, prospects of bioinspired flexible electronics in fundamental neuroscience and clinical applications are summarized.

## 2. Bioinspired flexible electronics for electrophysiological recording

Over the past decades, various bioinspired flexible electronics have been developed, including fibre neural electrodes that mimic the structures of neurons or neuronal processes^21,22^ and mesh/grid neural electronics^23,24^ that mimic the structures of neural networks in the brain. Bioinspired fibre electrodes with cellular/subcellular feature size and tissue-like mechanical stiffness can lead to greatly reduced tissue damage during implantation and minimized inflammatory response over chronic studies. On the other hand, the macroporous structures of mesh electronics have been shown to promote neuron interpenetration and the formation of seamless probe–tissue interfaces. Bioinspired flexible electronics that interface synthetic tissues, spheroids/organoids, and *in vivo* brain tissues will be discussed below.

### 2.1 Interfaces between bioinspired flexible electronics and synthetic tissues

**Fig. 1:**
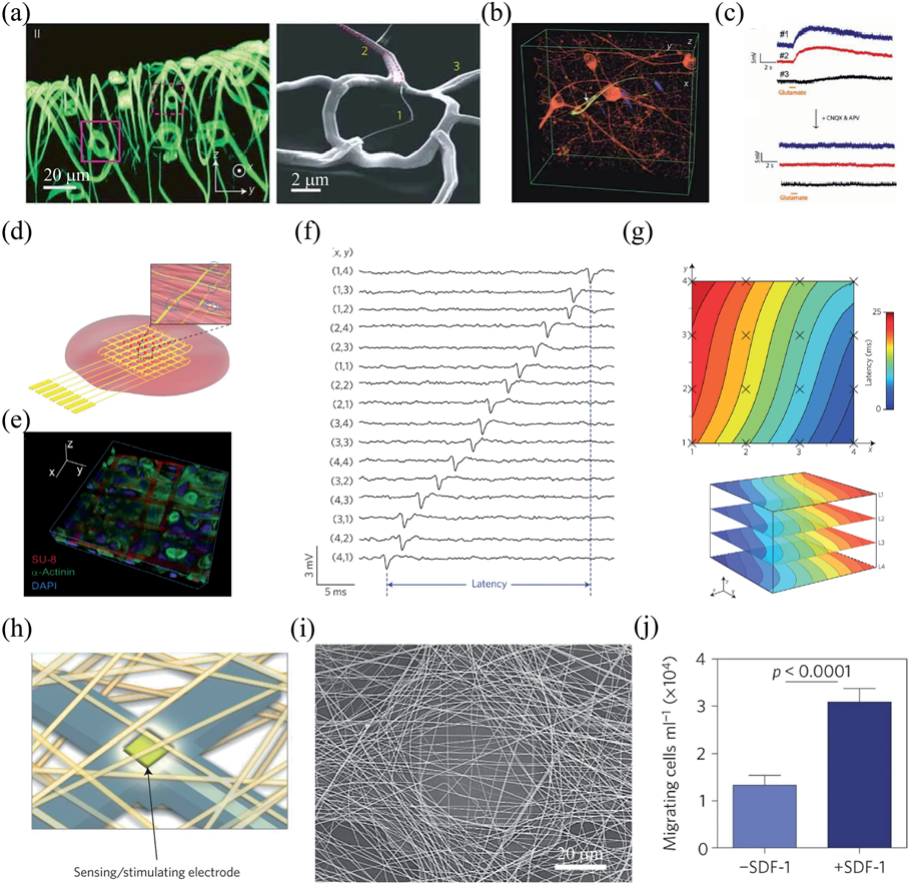
Interfaces between bioinspired flexible electronics and synthetic tissues. (a) Morphology of a highly curvilinear and porous nanoES. (b) The intimate nanoES–neural interface with an interpenetrating neurite. (c) Multiplexed LFP recording with the nanoES/neural hybrids. (a)–(c) have been reproduced from ref. 27 with permission from Nature Publishing Group, copyright 2012. (d and e) Hybrids of cardiac tissues and multilayer silicon nanowire FET array scaffolds. (f) Multiplexed action potential recording with the silicon nanowire FET array. (g) Mapping of the action propagation through a 3D cardiac tissue. (d)–(g) have been reproduced from ref. 30 with permission from Nature Publishing Group, copyright 2016. (h) Schematic of the microelectronic cardiac hybrids and their applications in tissue function sensing and regulation. (i) A recording/stimulating electrode covered with polymers. (j) Stromal cell-derived factor-1 releasing induced cell migration promotion. (h)–(j) have been reproduced from ref. 31 with permission from Nature Publishing Group, copyright 2016.

Conventional flexible electronics are mostly planar devices that can be used as 2D substrates for cell cultures.^25,26^ However, 2D cell cultures are generally insufficient to remodel cell–cell and cell–extracellular matrix interactions in 3D *in vivo* conditions. Bioinspired flexible electronics provide a promising platform for highly compatible and 3D cell cultures. As an example, Tian *et al.* developed a 3D nanoelectronic scaffold (nanoES) to mimic the structure of extracellular matrix.^27^ The nanoES exploited silicon nanowire field effect transistors (FETs) as sensing units and had a highly curvilinear and porous structure (Fig. 1a). The nanoES were biocompatible and could be readily incorporated with different macroporous biomaterials for 3D cultures of a variety of cells, including neurons, cardiomyocytes, and smooth muscle cells, to yield 3D nanoES/tissue hybrids. The macroporous structure of the nanoES allowed the interpenetration of the interior cells, such as embryonic rat hippocampal neurons, to form a 3D and seamless probe–tissue interface (Fig. 1b). The intimate nanoelectronic–tissue interfaces enabled stable 3D recording of local field potentials (LFPs) in response to the addition of glutamate or synaptic blockers (Fig. 1c). In another study, Cools *et al.* fabricated 3D microelectrode arrays through a residual stress induced self-folding process.^28^ Compared to devices with planar and open configuration, the 3D microelectrode arrays exhibited much tighter electrode–cell interface and thus achieved a twofold higher signal amplitude when interfacing with cardiomyocytes. In addition, Yan *et al.* developed a 3D bilayer electronic cage, which guided the growth of dorsal root ganglion cells into networks after a 35 day culture.^29^

Bioinspired flexible electronics provide an attractive platform for 3D recording and manipulation of cellular activities. For example, Duan *et al.* fabricated a folded, 16-channel silicon nanowire FET array as nanoelectronic scaffolds for 3D cell cultures (Fig. 1d).^30^ Neonatal rat ventricular cardiomyocytes seeded and cultured within the nanoelectronic scaffold could grow into nanoeletronics-innervated tissues in 7 days (Fig. 1e). The cultured cardiomyocytes were in close contact and aligned to the nanoelectronic scaffold. The intimate interface between the nanoelectronics and cardiomyocytes enabled reliable multi-channel action potential recording (Fig. 1f). In addition, the nanowire FET array in the scaffold allowed quantitative mapping of the action potential propagation across the 3D cardiac tissues (Fig. 1g). Feiner *et al.* integrated a porous electronic mesh with drug-containing polymers for simultaneous activity recording and modulation (Fig. 1h and i).^31^ The drugs in the polymers could be spatially released by electrical stimulation to regulate tissue functions. For example, under electrical stimulation, the release of stomal cell-derived factor-1 (SDF-1) could recruit bone marrow-derived stem cells and progenitor cells, and promote the cell migration *in vitro* (Fig. 1j).

### 2.2 Bioinspired flexible electronics interfacing spheroids and organoids

**Fig. 2:**
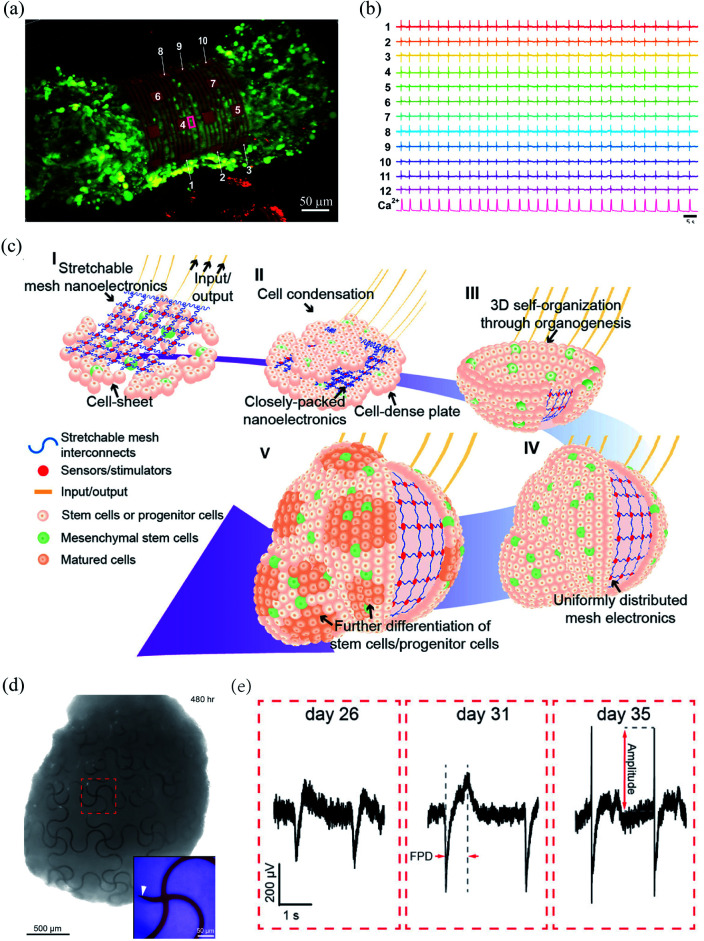
Bioinspired flexible electronics interfacing spheroids and organoids. (a) A CM spheroid encapsulated by the microelectrode array. (b) Microelectrode array for multiplexed LFP recording of the CM spheroid. (a) and (b) have been reproduced from ref. 34 with permission from American Association for the Advancement of Science, copyright 2019. (c) Integration of mesh electronics into organoids through organogenesis. (d) Mesh electronics distributed uniformly in a human cardiac organoid with seamless device–tissue interfaces. (e) Evolution of LFPs of cardiomyocyte cells during organogenesis. (c)–(e) have been reproduced from ref. 35 with permission from American Chemical Society, copyright 2019.

Spheroids and organoids have the potential to recapitulate complex features of *in vivo* 3D microenvironments.^32,33^ Therefore, they are widely applied to model human development and pathology. Kalmykov *et al.* developed a 3D self-rolled gold microelectrode array with a feature size of 25 μm × 25 μm to interface human embryonic stem cell-derived cardiomyocyte (CM) spheroids.^34^ The self-rolling process was induced by the residual mismatch stress between the polymer supporting layer and the above metal layers (Fig. 2a). The recording electrodes were coated with a layer of (3,4-ethylenedioxythiophene)/poly(sodium 4-styrenesulphonate) (PEDOT:PSS), which reduced their impedance from 0.56 ± 0.25 MΩ to 14 ± 7.6 kΩ at 1 kHz. Owing to the tight electrode–CM organoid interface, the microelectrode array could stably record the activities of the CM spheroid with a high signal-to-noise ratio of *ca.* 9 (Fig. 2b).

Li *et al.* recently developed a 3D assembly method to integrate mesh electronics across an entire organoid by cell–cell attraction forces during organogenesis.^35^ The fabrication of the 3D assembly, termed cyborg organoid, started from the integration of mesh electronics with a coculture of human mesenchymal stem cells and human-induced pluripotent stem cells. The human mesenchymal stem cells then initiated cell condensation, with the aggregation, proliferation, and migration of human-induced pluripotent stem cells, and the mesh electronics was embedded within the closely packed cells. The interwoven cell-mesh electronics layer then contracted and curled into a bowl geometry and finally into a spherical organoid/nanoelectronics hybrid (Fig. 2c). In order to accommodate the large volume change during organogenesis, the mesh electronics in the cyborg organoid was designed with a serpentine structure. This enabled an in-plane stretchability of up to 30% and an out-of-plane compressibility several times smaller than its initial volume. Due to the unique 3D self-organization process, the mesh electronics was embedded throughout the organoids with a uniform distribution (Fig. 2d). The mesh electronics could stably monitor the evolution of the field potentials of the cardiomyocyte cells during organogenesis because of their seamless interfaces (Fig. 2e).

### 2.3 Bioinspired flexible electronics for *in vivo* neural interfacing

*In vivo* neural recording has greatly advanced our understanding in neural circuit functions and promoted the development of clinical techniques for the treatment of neurological diseases. This section will discuss bioinspired flexible electronics, including fibre microelectrodes, mesh electronics, and epicortical grid electrodes for *in vivo* neural interfacing.

#### 2.3.1 Fibre microelectrodes

**Fig. 3:**
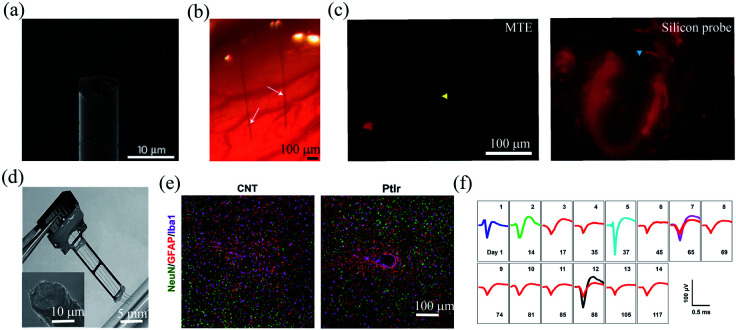
Carbon-based fibre microelectrodes for *in vivo* neural interfacing. (a) Scanning electron microscopy image of the ultrasmall carbon fibre microelectrode. (b) Optical image of two carbon fibre microelectrodes implanted into the rat cortex. (c) Comparison of bleeding caused by MTE probes and silicon probes. (a)–(c) have been reproduced from ref. 41 with permission from Nature Publishing Group, copyright 2012. (d) A four-channel CNT fibre microelectrode array. (e) Comparison of the tissue inflammatory responses to CNT fibre microelectrodes and PtIr electrodes. (f) Stable single-unit neural recording from day 1 to day 117 with an electrode made of a 15 μm diameter CNT fibre. (d)–(f) have been reproduced from ref. 50 with permission from American Chemical Society, copyright 2019.

The implantation of neural probes into the brain is a traumatic event that induces local perturbation to brain tissues, such as tissue displacement and vessel disruption.^36^ The acute tissue damage can initiate progressive inflammatory tissue responses. One strategy to reduce the inflammatory responses of the brain tissues is to coat the electrode surfaces with hydrophilic polymers, such as poly(ethylene glycol) (PEG),^37^ poly(2-hydroxyethyl methacrylate) (pHEMA)^38^ and zwitterionic polymers.^39^ These hydrophilic layers could significantly reduce the adsorption of proteins and inflammatory cells, and thus improve the chronic performance of the implanted electrodes. Another strategy to reduce the inflammatory response is to minimize the insertion size of the neural electrodes. Owing to their small cross-sectional footprints, fibre microelectrodes hold great promise to reduce acute implantation damage and inflammatory responses of the brain tissues.^40^ For example, Kozai *et al.* developed a ∼8.5 μm diameter carbon fibre composite microelectrode termed microthread electrode (MTE) (Fig. 3a).^41^ The MTE consisted of a 7 μm diameter carbon-fibre core, a 800 nm thick poly(*p*-xylyene) insulation layer, and a 200 nm thick poly(ethylene glycol) methacrylate anti-biofouling layer. In addition, a layer of PEDOT:PSS was deposited on the exposed tip for reduced impedance. The ultrasmall diameter enabled MTE probes to cause minimal insertion trauma, with limited bleeding around the probes, as compared to conventional silicon probes (Fig. 3b and c). Moreover, the MTE probes were shown to elicit greatly reduced glial accumulation and cell depletion than silicon probes. Therefore, they enabled stable single-unit activity recording in rat brain for over 5 weeks. In addition, carbon fibre electrode array with 16,^40,42^ 32,^43^ and 64 channels^44^ have been fabricated for multi-site neural activity recording from rats,^40,43,44^ and songbirds.^42^ Although PEDOT:PSS coating can effectively reduce the electrode impedance, it tends to degrade slowly over time and results in recording instability. Patel *et al.* found that the chronic stability of the electrode could be greatly improved by using PEDOT:sodium *p*-toluenesulfonate as the coating layer.^40^ They further show that PEDOT:sodium *p*-toluenesulfonate coated electrode array allowed stable single-unit recording from rat motor cortex for over 3 months.

Carbon nanotubes (CNTs) have also been studied as electroactive materials for implantable neural electrodes due to their large surface areas and good conductivity.^45–51^ Over the past decade, a variety of fibre neural probes have been fabricated using CNTs or their nanocomposites.^46–51^ For example, Vitale *et al.* fabricated microelectrodes from wet-spun CNT fibres with polystyrene–polybutadiene as insulation layer.^47^ Owing to the large surface areas of CNT fibres, the impedance of the microelectrodes was 2.5 to 6 times lower than those of tungsten and carbon fibres. Moreover, CNT fibres exhibited orders magnitude lower stiffness than conventional PtIr electrodes. As a result, they enabled stable single-unit recording from the rat primary motor cortex for over 4 weeks. Recently, Lu *et al.* fabricated CNT fibre microelectrodes through dry-spinning of vertically aligned double and triple-walled CNT forest and a further insulation process with parylene C.^50^ Nitric acid-treated CNT fibres exhibited an impedance of 41.95 ± 3.62 kΩ, which was 9 times lower than that of PtIr electrodes of a similar size. Moreover, they exhibited a bending stiffness that was approximately three orders of magnitude lower than that of PtIr electrodes and thus elicited much reduced inflammatory responses in brain tissues (Fig. 2e). As a result, the CNT fibres allowed stable recording of spontaneous single-unit spikes from the thalamus of anesthetized rats for over 4 months (Fig. 2f).

**Fig. 4:**
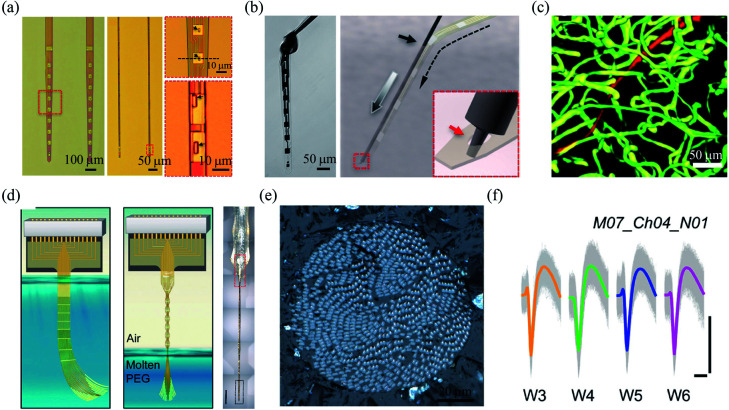
Polymer based-fibre microelectrodes for *in vivo* neural interfacing. (a) Structures of NET-50 and NET-10 probes. (b) A knotted NET-50 probe with high flexibility and robustness (left), and schematic of the NET engaging mechanism (right). (c) The intact microvessels around a NET-10 probe. (a)–(c) have been reproduced from ref. 21 with permission from American Association for the Advancement of Science, copyright 2017. (d) Schematic of the self-assembly of Neurotassel in molten PEG and an assembled Neurotassel/PEG composite fibre with 16 electrodes. Scale bar, 500 μm. (e) Scanning electron microscopy image of the Neurotassel/PEG fibre with 1024 electrodes. (f) Stable tracking of the same neuron from 3 to 6 weeks. Scale bars, 100 μm (vertical), 1 ms (horizontal). (d)–(f) have been reproduced from ref. 22 with permission from American Association for the Advancement of Science, copyright 2019.

Compared to rigid materials, polymers have several orders of magnitude lower Young's moduli, and thus can allow the fabrication of ultraflexible fibre microelectrodes.^52^ For example, Luan *et al.* developed ultraflexible SU-8 based nanoelectronic thread (NET) brain probes with gold pads as the recording electrodes.^21^ They designed two types of probes named NET-50, and NET-10, with the smallest cross-section of only 10 μm × 1.5 μm (Fig. 4a). The bending stiffness of the NET probes was only ∼10^−15^ N m^2^, which resulted in an extremely low probe–tissue interfacial force of nanonewton range. The NET probes were delivered into brain tissues with the assistance of an ultrathin carbon fibre or tungsten wire, with an overall insertion footprint of only *ca.* 10 μm (Fig. 4b). As a result, the NET probes induced cellular-sized surgical damage to the brain tissue, and minor blood–brain-barrier leakage was observed at the implantation site (Fig. 4c). Moreover, the NET probe was highly biocompatible, and no chronic tissue response was observed at 5 months postimplantation. Owing to the seamless device–tissue interface, individual neurons from mouse somatosensory cortex were stably tracked by the NET probes for over 4 months. In another work, they further reduced the footprint of the NET probes to a cross-sectional area of only 0.8 μm × 8 μm.^53^ The small lateral dimensions of the NET probes allowed for simultaneous implantation of seven probes with an inter-probe spacing of only 60 μm.

Brain functions involve the coordinated activity of large populations of neurons.^54^ Therefore, it is essential to develop high-density neural probes that can simultaneously record the activity of a large number of neurons. Recently, our group developed a high-density filament neural probe termed Neurotassel.^22^ The Neurotassel had a unique plane-mesh-filament structure design, and the total thickness of the probe was only 1.5 to 3 μm. As a result, the filament electrodes exhibited a small bending stiffness of only <0.1 nN m. When the flexible filaments were withdrawn from a molten PEG bath, they spontaneously self-assembled into a stiff fibre through elastocapillary interactions (Fig. 4d). Notably, the Neurotassel/PEG fibre assembled from a 1024-channel device had a diameter of only ∼100 μm (Fig. 4e). The cross-section of each filament electrode was only 3 μm × 1.5 μm, approaching sizes of single neurites. The Neurotassel/PEG fibre could be directly inserted into the targeted brain regions of mice, where PEG was gradually dissolved to release the filament electrodes for neural activity recording. Due to the high flexibility and small footprint of the filament electrodes, Neurotassels elicited minimal neuronal cell loss around the implantation sites and allowed stable activity tracking of the same neurons in mouse medial prefrontal cortex for 6 weeks (Fig. 4f).

#### 2.3.2 Intracortical mesh electronics

**Fig. 5:**
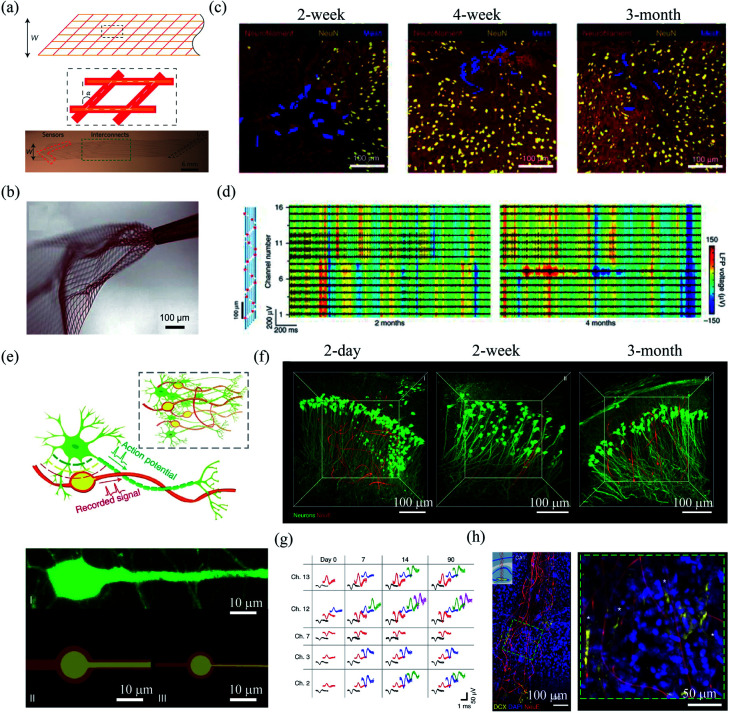
Mesh electronics for *in vivo* neural interfacing. (a) Structure of mesh electronics. (b) Unfolding of mesh electronics after the injection. (a) and (b) have been reproduced from ref. 55 with permission from Nature Publishing Group, copyright 2015. (c) The interface between injected mesh electronics and brain tissue after 2 week, 4 week and 3 month postimplantation. (d) Mesh electronics for stable LFP recording 2 months and 4 months postimplantation. (c) and (d) have been reproduced from ref. 59 with permission from Nature Publishing Group, copyright 2016. (e) NeuE with neuron-mimetic structures. (f) 3D interfaces between NeuE and neurons 2 day, 2 week, and 3 month postimplantation. (g) Stable single-unit activity recording with NeuE over 3 month postimplantation. (h) The migration of neural progenitor cells along NeuE. (e)–(h) have been reproduced from ref. 62 with permission from Nature Publishing Group, copyright 2019.

In order to form a 3D and seamlessly integration with the brain tissue, Liu *et al.* developed a free-standing and macroporous mesh electronics with submicrometer thickness.^55^ The mesh electronics consisted of exposed silicon nanowire FETs or platinum electrodes, SU-8 sandwiched gold interconnects, and input/output pads in the longitude direction and SU-8 scaffolds in the transverse direction (Fig. 5a). The input/output pads can be connected to the external recording instruments through conductive ink printing,^56^ plug-and-play,^57^ or capillary-force-induced deformation.^58^ The mesh electronics was highly flexible, with a bending stiffness comparable to that of a 150 μm-thick brain tissue.^16,17^ Notably, the ultraflexible mesh electronics can be constrained in a capillary needle of only hundreds of micrometers in diameter and allowed for implantation by syringe-injection (Fig. 5b). As a result, the mesh electronics could be precisely injected into targeted regions in mice brain with a spatial precision of *ca.* 20 μm.^56^ Chronic studies showed that the mesh electronics formed a seamless interface with the brain tissue. Fu *et al.* found that mesh electronics produced little inflammation and the neurons at the probe–brain interface maintained natural distribution for months-to-year timescale (Fig. 5c).^59^ By contrast, polymer film-based planar probe elicited substantial accumulation of astrocytes and microglia, as well as a 20 to 50 μm neuron depletion region around the probe.^17^ They further demonstrated that 16-channel mesh electronics with 20 μm diameter platinum electrodes allowed stable LFP recording of awake mouse at 2 and 4 months postimplantation (Fig. 5d). In a following study, they injected 4 of 32-channel mesh electronics into mouse motor cortex and somatosensory cortex/hippocampus and achieved stable recording from different cortical areas.^60^ In another study, Hong *et al.* adopted mesh electronics to record the activity of single retinal ganglion cells of awake mouse.^61^ The retinal ganglion cells were found to show stable responses to visual stimuli of air puffs, brightness, and moving gratings.

In order to further reduce the disturbance of endogenous neural networks in the brain, Yang *et al.* developed bioinspired neuron-like electronics termed NeuE.^62^ The feature sizes of the platinum electrode (10 μm and 8 μm) and gold interconnects in the NeuE (1 μm and 0.6 μm) were similar to those of the soma and neurite of a typical pyramidal neuron (Fig. 5e). The NeuE exhibited a bending stiffness of only ∼10^−16^ N m^2^. In addition, the reduced feature size also resulted in a low filling fraction of only 0.07–0.3%, which minimized cell exclusion induced by probe occupying. Therefore, the NeuE allowed the intimate interpenetration of neurons to form a structurally and functionally stable device–brain interface. They showed that neurons could form seamless integration with the implanted NeuE (Fig. 5f). Owing to the minimal neuron disturbance, the 16-channel mesh electronics allowed stable tracking of the same populations of neurons in different mouse cortical areas for 3 months (Fig. 5g). More importantly, the NeuE could promote the migration of endogenous neural progenitor cells to form new neurons along the scaffolds (Fig. 5h), which opens up new opportunities for transplantation-free regeneration.

#### 2.3.3 Epidural grid electrodes

**Fig. 6:**
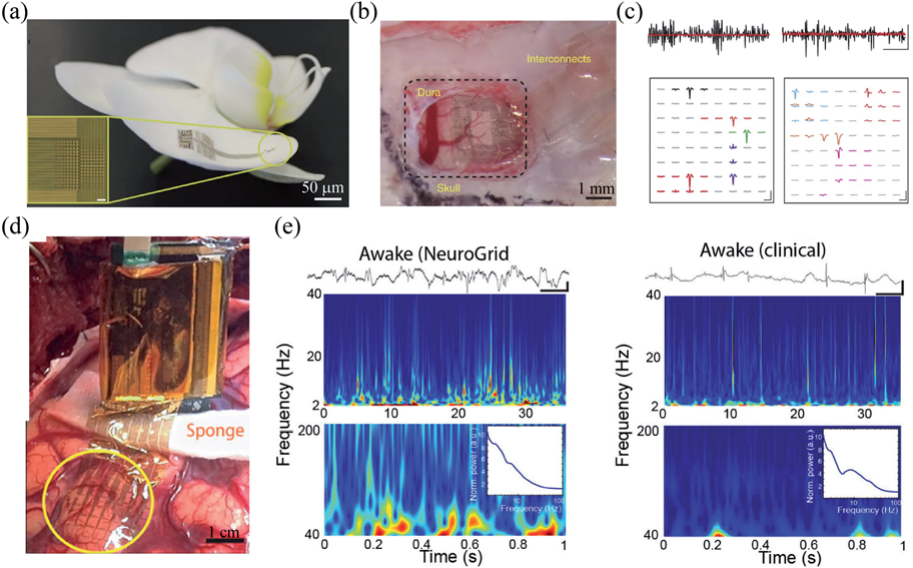
Epidermal grid electrodes for *in vivo* neural interfacing. (a and b) The Neurogrid conformably attaches on an orchid petal and a rat cortical surface. (c) Extracellular action potential recording in cortex (left) and hippocampus (right) of a rat with the Neurogrid. Scale bars (top): 10 ms, 50 μV. Scale bars (bottom): 1.5 ms, 50 μV. (a)–(c) have been reproduced from ref. 24 with permission from Nature Publishing Group, copyright 2015. (d) A 240-channel Neurogrid conformably adheres to the cortical surface of an epilepsy patient. (e) Comparison of the performance between the Neurogrid and clinical strips. Scale bars: 1 s, 500 μV. (d) and (e) have been reproduced from ref. 65 with permission from American Association for the Advancement of Science, copyright 2016.

Comparing to intracortical electrodes, electrocorticography or epidural electrodes can record neural activity from the surface of the brain and cause minimal physiologic disruption to the brain tissue. Moreover, epidural electrodes can allow activity recording from a large volume of neurons. Therefore, they are widely used in clinical diagnosis of neurological disorders, such as epilepsy, tumors, and vascular abnormalities.^63^ An intimate electrode–tissue interface is crucial for the stable and reliable recording of the neural activity.^64^ Khodagholy *et al.* developed an ultracomfortable epidural electrode array termed Neurogrid, which exploited a 4 μm thick parylene film as the supporting substrate.^24,65,66^ Owing to its subcellular thickness, the Neurogrid was highly flexible and could be comfortably adhered to the curvilinear surfaces of an orchid petal (Fig. 6a). Moreover, recording sites was similar to that of the neurons in rat brain. Notably, the ultraconformal electrode–tissue interface and the cellular feature size of the Neurogrids allowed stable single-unit recording of action potentials from superficial cortical neurons of rats for over 10 days (Fig. 6b and c). In addition, the Neurogrids could also be applied to record LFPs and action potentials from epilepsy patients.^65^ Owing to its high flexibility and hydrophobic surface, the Neurogrid formed a conformal and stable interface with the cortical surface despite brain pulsations (Fig. 6d), which enabled a comparable performance in interictal epileptiform discharge recording with clinical strip electrodes and provided more information in high-frequency region (Fig. 6e).

## 3. Conclusions and prospects

In this review, we discussed recent development of bioinspired flexible electronics, including fibre microelectrodes, mesh electronics, and grid electrodes. The tissue-like mechanical stiffness and cellular/subcellular feature sizes of bioinspired flexible electronics enabled the formation of a seamless device–tissue interfaces and thus stable chronic neural recording. Due to the tissue-like bending stiffness, current bioinspired flexible neural probes mostly require the assistance of rigid probes^21,46,47,49–51^ or syringe needles^17,55–62^ for implantation. The introduction of these temporary carriers inevitably increases the total footprint of the probes, which aggravates the acute injury to the brain tissue. Although flexible neural probes could also be temporarily stiffened by forming composites with polymers, polymers should be carefully chosen for not introducing inflammatory responses to the brain tissue. New strategies that can allow the implantation of flexible neural probes without increasing their footprint are highly desirable. Recently, Vitale *et al.* used the microactuation of a microfluidic device to assist the implantation of the fibre microelectrode and a 22 μm diameter CNT fibre electrode was directly inserted into the rat brain.^48^ Our group recently exploited magnetic actuation to assist the insertion of flexible filament probes into mouse brain.^67^ These methods can potentially facilitate the minimal invasive implantation of flexible neural probes. In addition, current bioinspired flexible neural probes are mostly designed to perform a single function, either for electrophysiological recording or electrical stimulation. Multifunctional neural probes with the capability to interfere with tissues for on-demand regulation of their function and assembly behaviour could greatly facilitate the tissue/organ recovery and regeneration. Recent examples of nanoelectronics promoting neural progenitor cells migration^62^ and nanoelectronics controlling cardiac tissue function regulation^31^ have opened up a variety of new opportunities for nanoelectronics-assisted tissue/organ recovery and regeneration. In addition, combining bioinspired flexible neural probes with optogenetic techniques can allow simultaneous long-term recording and targeted stimulation of neuronal activity, thus opening up new opportunities for the study of neural circuit functions. Finally, current animal models for bioinspired neural probes are limited to rodents. Future studies that integrate flexible neural probes with human brain organoids could improve our understandings of human brain development and facilitate the discovery of new treatment strategies for human brain diseases.^68^ Capitalizing on these opportunities will require coordinated efforts across many disciplines, including material science, electronics, mechanical engineering, and neuroscience.

## Conflicts of interest

There are no conflicts to declare.

## Supplementary Material
